# Effects of Radiographic Contrast Media on the Micromorphology of the Junctional Complex of Erythrocytes Visualized by Immunocytology

**DOI:** 10.3390/ijms150916134

**Published:** 2014-09-12

**Authors:** Ralf-Peter Franke, Anne Krüger, Tim Scharnweber, Folker Wenzel, Friedrich Jung

**Affiliations:** 1 Department of Biomaterials, University of Ulm, 89081 Ulm, Germany; E-Mail: rp.franke@web.de; 2 Institute of Biomaterial Science and Berlin-Brandenburg Center for Regenerative Therapies, Helmholtz-Zentrum Geesthacht, 14513 Teltow, Germany; E-Mail: anne.krueger@hzg.de; 3 Institute for Biological Interfaces, Karlsruhe Institute of Technology (KIT), 76344 Eggenstein-Leopoldshafen, Germany; E-Mail: tim.scharnweber@kit.edu; 4 Institute for Transplantation Diagnostics and Cell Therapeutics, Medical Center of University, 40225 Düsseldorf, Germany; E-Mail: folker.wenzel@hs-furtwangen.de

**Keywords:** radiographic contrast media, Iopromide, Iodixanol, erythrocytes, cytoskeleton, band 3, band 4.9, actin

## Abstract

Effects of radiographic contrast media (RCM) application were demonstrated *in vitro* and *in vivo* where the injection of RCM into the A. axillaris of patients with coronary artery disease was followed by a significant and RCM-dependent decrease of erythrocyte velocity in downstream skin capillaries. Another study in pigs revealed that the deceleration of erythrocytes coincided with a significant reduction of the oxygen partial pressure in the myocardium—supplied by the left coronary artery—after the administration of RCM into this artery. Further reports showed RCM dependent alterations of erythrocytes like echinocyte formation and exocytosis, sequestration of actin or band 3 and the buckling of endothelial cells coinciding with a formation of interendothelial fenestrations leading to areas devoid of endothelial cells. Key to morphological alterations of erythrocytes is the membrane cytoskeleton, which is linked to the band 3 in the erythrocyte membrane via the junctional complex. Fundamental observations regarding the cell biological and biochemical aspects of the structure and function of the cell membrane and the membrane cytoskeleton of erythrocytes have been reported. This review focuses on recent results gained, e.g., by advanced confocal laser scanning microscopy of different double-stained structural elements of the erythrocyte membrane cytoskeleton.

## 1. Introduction

In 1985 Dawson described morphological alterations of erythrocytes after contact with radiographic contrast media (RCM) which he attributed to chemotoxic effects [1]. It is still unclear which mechanisms underlie the described cellular alterations like echinocyte formation [2,3,4,5,6], exocytosis and the buckling of endothelial cells coinciding with a formation of interendothelial fenestrations often leading to cell free, denuded areas [7,8,9]. Effects of RCM application were demonstrated *in vitro* and *in vivo* where the injection of RCM into the A. axillaris of patients with coronary artery disease was followed by a significant and RCM-dependent decrease of erythrocyte velocity in downstream skin capillaries [10,11]. A following study in pigs revealed that the deceleration of erythrocytes coincided with a significant reduction of the oxygen partial pressure in the myocardium—supplied by the left coronary artery—following the administration of RCM into this artery [12]. The varying extent of the morphological alterations observed *in vitro* was well correlated to varying deteriorations of the local microcirculation *in vivo*. Since heart rate, cardiac output and blood pressure practically remained constant it can be assumed that the local deterioration of the microcirculation was mainly based on the morphological alterations of erythrocytes and endothelial cells in the microcirculation. Plasma viscosity, too, influences the capillary perfusion [13]. Since the viscosities of RCM are much higher than that of blood plasma, there is a clear increase of viscosity of the blood plasma/RCM mixture in the downstream microcirculation after arterial injection of RCM—depending on the duration of the injection and the amount of RCM applied. There were differences in the capillary perfusion clearly due to differences in RCM viscosity, but these became obvious only after a RCM was applied (Iopentol, η = 1.7 mPa**^.^**s) with a viscosity nearly as low as the plasma viscosity [11]. Those RCM mostly used in coronary angiography have viscosities which do not differ enough from one another—η varying between 7 and 11 mPa**^.^**s—and hence do not suggest to attribute the RCM-dependent strongly differing morphological and pathophysiological effects to the variation in RCM viscosity. This became obvious by the strong morphological differences in echinocyte formation and endothelial cell buckling *in vitro* after addition of Iopromide to plasma or cell culture media, while the addition of Iodixanol did not affect the cells negatively. Since both RCM have similarly elevated viscosities it is assumed that the different morphological changes observed seem to be responsible for the microcirculatory deteriorations.

Endothelial cell buckling—the cell thickness increased after application of Iopromide for almost 50%, with practically no increase in cell thickness after Iodixanol application [7]—in conjunction with the echinocyte formation [4]—which is accompanied by a marked rigidification of the erythrocytes [14,15]—were assumed to be the reason for the short-termed cessations of blood flow in the local microcirculation [16].

Key to morphological alterations of erythrocytes is the membrane cytoskeleton, which is linked to the band 3 (AE1) in the erythrocyte membrane via the junctional complex. Basic results about the cell biology and biochemistry [17,18,19] of structure and function of the cell membrane and the membrane cytoskeleton of erythrocytes have recently been characterized further using immunocytochemical methods for the double staining of different elements of the membrane cytoskeleton [20,21,22,23].

## 2. Amount of Echinocyte Formation in Plasma Supplemented with Radiographic Contrast Media (RCM)

**Figure 1:**
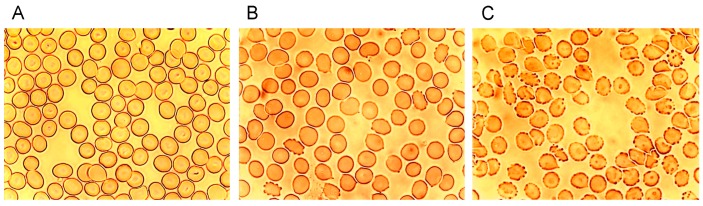
Radiographic contrast media (RCM)-induced echinocyte formation (reprinted from reference [6], Copyright 2008, with permission from IOS Press); (**A**) Erythrocytes resuspended in autologous plasma (40% *v*/*v*); (**B**) Erythrocytes resuspended in an Iodixanol/plasma mixture (40% *v*/*v*); (**C**) Erythrocytes resuspended in an Iopromide/plasma mixture (40% *v*/*v*).

Figure 1 shows microphotographs taken in a transmission light microscope at a primary magnification of 1:40 of the echinocyte formation after suspension of erythrocytes in autologous plasma (Figure 1A), in an Iodixanol320/plasma mixture (40% *v*/*v*; Figure 1B) and in an Iopromide350/plasma mixture (40% *v*/*v*; Figure 1C). This concentration was chosen based on an estimate of the RCM concentration in the vasculature after a bolus injection of RCM in an artery [24,25].

While in autologous plasma 4.6% ± 4.5% echinocytes were found, 24.3% ± 29.6% of echinocytes appeared after adding Iodixanol and this number increased to 80.6% ± 17.4% of echinocytes after addition of Iopromide [6]. This study revealed a significantly higher number (3.3-fold) of echinocytes after Iopromide. Table 1 shows the percentages of echinocytes in different stages according to Bessis [26].

## 3. Stages of Echinocyte Formation in Plasma Supplemented with RCM

Table 1 shows that not only the numbers of echinocytes were significantly higher after incubation of erythrocytes in an Iopromide/plasma mixture but also the stages according to Bessis differed significantly. While there were only very few stage II echinocytes and no stage III echinocytes at all in the Iodixanol/plasma mixture, there were 21.7% stage II echinocytes and 0.53% stage III echinocytes in the Iopromide/plasma mixture.

**Table 1:** Bessis stages in (%) of echinocytes suspended in autologous plasma, in an Iodixanol/plasma mixture (40% *v*/*v*), and in an Iopromide/plasma mixture (40% *v*/*v*).

Bessis Stages	Echinocytes in Autologous Plasma	Echinocytes in an Iodixanol/Plasma Mixture	Echinocytes in an Iopromide/Plasma Mixture
Stage I	4.1 ± 4.5	22.2 ± 19.9	58.4 ± 10.0
Stage II	0.46 ± 1.13	1.90 ± 3.05	21.7 ± 13.5
Stage III	0 ± 0	0 ± 0	0.53 ± 0.93

## 4. Reversibility of Echinocyte Formation after Contact with RCM

**Figure 2:**
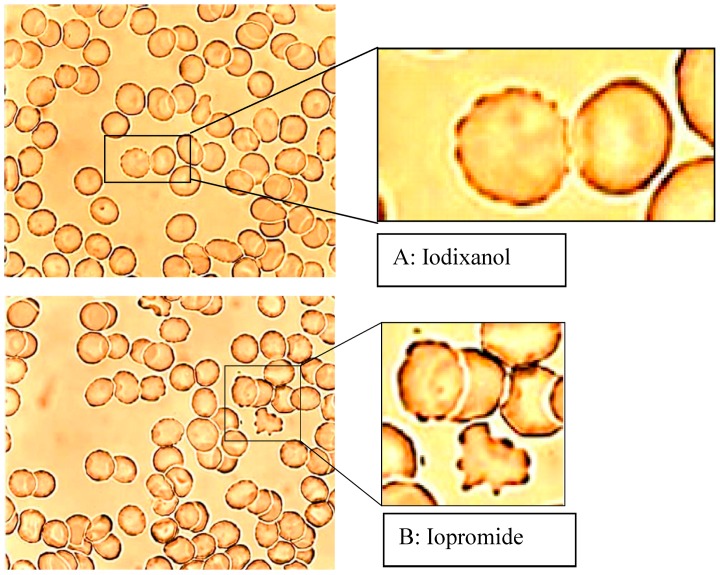
Erythrocytes in an Iodixanol/plasma mixture (**A**) or Iopromide/plasma mixture (**B**) 40% *v*/*v* each after resuspension in autologous RCM-free plasma (modified according to reference [6], Copyright 2008, with permission from IOS Press).

The examination of the reversibility of the echinocyte formation by resuspension of echinocytes in autologous plasma, after they had been suspended in RCM/plasma mixture in a previous step, showed that only erythrocytes initially exposed to Iopromide remained in higher stages according to Bessis, particularly in stage III (see Figure 2, [6]).

Therefore, it is important to note, that different RCM (e.g., Iopromide *versus* Iodixanol) had a different influence on echinocyte formation, first on the total number of echinocytes, second on the different stages of echinocytes and third on differences in the reversibility of echinocytes to discocytes.

## 5. Membrane Cytoskeleton of Human Erythrocytes in Autologous Plasma

Changes of erythrocyte morphology are effected by their membrane cytoskeleton. Backbone of the membrane cytoskeleton are tetramers of heterodimeric α-, β-spectrins usually found in a pentagonal or hexagonal configuration [27,28]. Actin is beside spectrin a main component of the membrane cytoskeleton [27] and seems to be an essential component together with other elements in a complex linking the membrane cytoskeleton to the erythrocyte cell membrane. Immunocytological double staining enabled the visualization of the distributions of the cytoskeletal spectrin and actin at the same time in identical cells [20].

**Figure 3:**
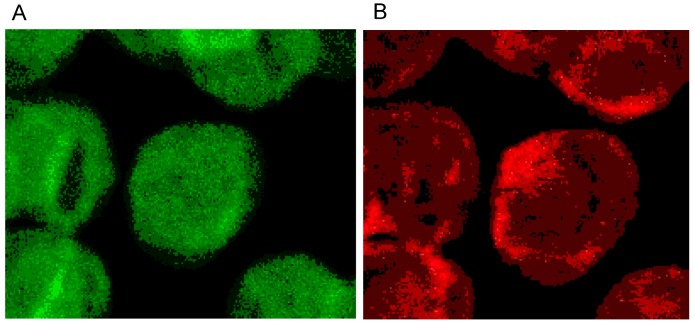
(**A**) Spectrin and (**B**) Actin (stained with antibody against β-actin) distribution in erythrocytes in autologous plasma (reprinted from reference [20], Copyright 2013, with permission from IOS Press) (Primary magnification: 1:63; zoom factor: 5).

The single erythrocyte in the center of Figure 3A displays the typical homogeneous spectrin distribution found in human erythrocytes incubated in autologous plasma, with slightly enhanced stain intensity at the cell rim. Contrary to the spectrin distribution, the distribution of actin was clearly non-homogenous showing strongly enhanced stain intensity at the cell rim. It was assumed that this might be due to elevated stresses in strongly curved cell regions [29].

**Figure 4:**
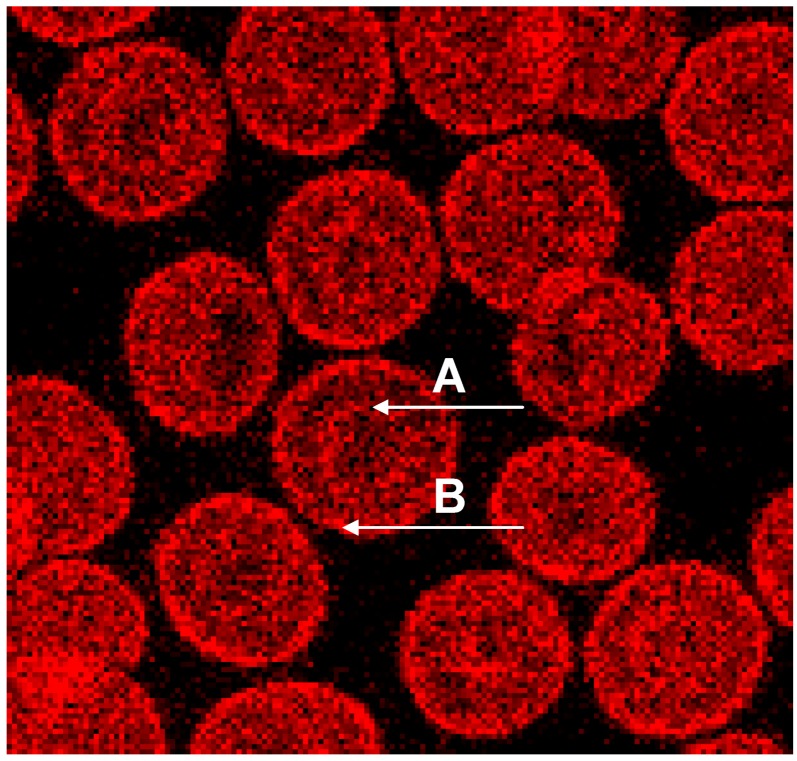
Phalloidin–Rhodamin stained actin in erythrocytes after suspension in autologous plasma; “A” marked arrow pointing at the periphery of the central dip, “B” marked arrow at the cell rim (reprinted from reference [23], Copyright 2013, with permission from IOS Press) (Primary magnification 1:63, zoom factor: 5).

Phalloidin–Rhodamin interacts with and allows us to visualize actin polymers assumed to consist of at least 16 monomers. It was thought that there is almost no highly polymerized actin in sessile discocytes [27,28]. Phalloidin–Rhodamin stained actin (see Figure 4) revealed that longer actin polymers occurred in cell regions characterized by prominent curvatures: these are the cell rim (see arrow B in Figure 4) and the periphery of the central cell dip (see arrow A in Figure 4). The distribution of all the actin revealed by the use of an antibody against β-actin (see Figure 3B) differed strongly from the actin distribution assessed by Phalloidin–Rhodamin.

It should be considered that “Fluorescence Microscopy” as applied earlier, usually did not grant images as rich in contrast as those produced nowadays applying “Confocal Laser Scanning Microscopy”. It can be assumed, therefore, that there are some larger actin polymers already in discocytes.

**Figure 5:**
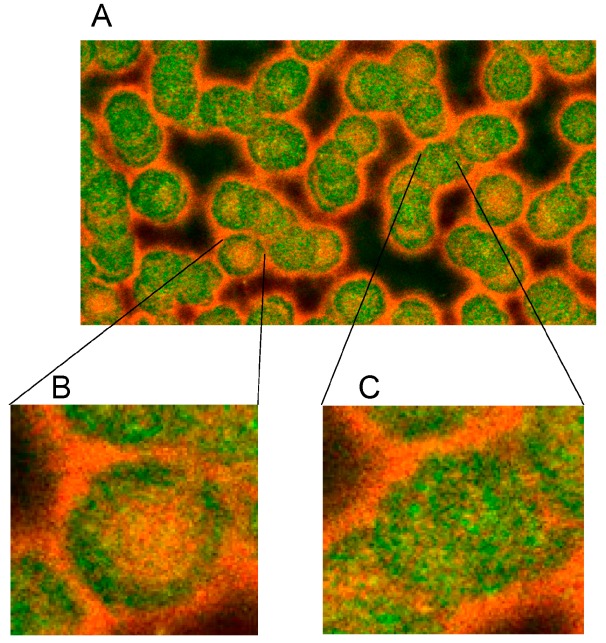
Merger of red (actin) and green (spectrin) channels of the confocal microscope showing erythrocytes after examination in autologous plasma (**A**–**C**) with details of magnified single (**B**) or aggregated erythrocytes (**C**) (reprinted from reference [20], Copyright 2013, with permission from IOS Press) (**A**) primary magnification 1:63; zoom factor 2.5; (**B**) primary magnification 1:63; zoom factor 5; (**C**) primary magnification 1:63; zoom factor 5.

The merger of red (actin) and green (spectrin) channels of the confocal microscope revealed that the spectrin-/actin-distribution in singularized erythrocytes differed from the distribution in aggregated discocytes (see Figure 5). As main aspect in aggregated erythrocytes spectrin appeared to be the more or less homogeneously distributed over the whole cell (see Figure 5C), whereas in singularized erythrocytes the red stained actin in central parts of cells was a noticeable aspect (see Figure 5B).

**Figure 6:**
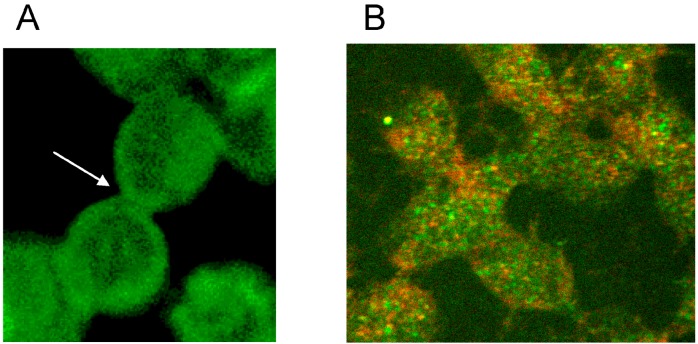
Spectrin reorganization in aggregated erythrocytes suspended in autologous plasma (reprinted from reference [20], Copyright 2013, with permission from IOS Press) (**A**) spectrin-stained erythrocytes; (**B**) Merger of red and green channels of the confocal microscope of spectrin–actin double stained erythrocytes. (Primary magnification 1:63; zoom factor 3.5).

The spectrin stain in the green channel revealed that there is a reformation of the spectrin network in aggregating erythrocytes as more and denser spectrin stain appeared at the cell periphery and more or less visible bands of stained spectrin developed in central parts of the cells (see Figure 6A). In the contact zone of aggregating cells, the spectrin nets of adjacent cells seemed to get very near to one another.

Double staining disclosed that there were many red pixels (actin) in the contact zone of cells, which could be observed already after staining of actin (see Figure 3B). Aggregated cells sometimes gave the impression as if cell fusion occurred (see Figure 6A (white arrow) and B). This is thought to be less probable since the formation of erythrocyte aggregates is regarded to be reversible [30,31,32,33]. If there would be cell fusion, the consecutive reformation of the discocytic state of the cells would afford energy. This point cannot be solved at the moment. There were reports, however, that metabolically depleted erythrocytes, whose rate of energy conversion was reduced drastically, clearly aggregated less and were also less deformable [34].

This study revealed that erythrocyte aggregation is accompanied by a reorganization of the spectrin network [20]. It might be speculated that this could be the main reason for the limitation in erythrocyte life time of 120 days in the blood circulation. If energy would be needed for the constantly afforded deformation work, this would lead to ATP depletion and to a break-down of energy supply, resulting in a rigidification of the erythrocyte [35] and its elimination in the sinusoid vessels of the spleen [36].

## 6. Effects of RCM on the Membrane Cytoskeleton of Human Erythrocytes

**Figure 7:**
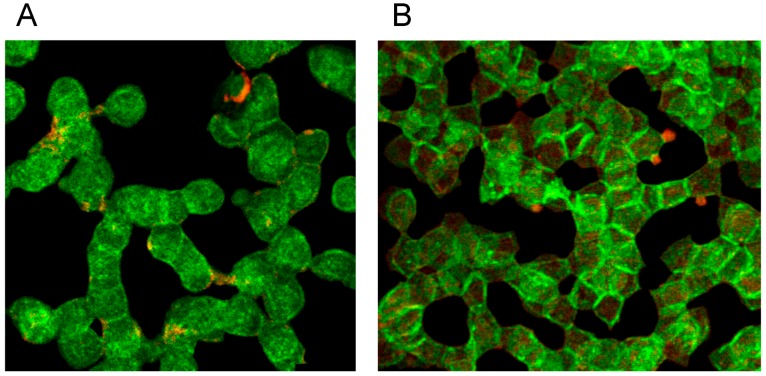
Spectrin reorganization in aggregated erythrocytes (reprinted from reference [20], Copyright 2013, with permission from IOSPress) (**A**) Merger of red (actin) and green (spectrin) channels of the confocal microscope after examination of spectrin–actin double stained erythrocytes suspended in plasma supplemented with Iodixanol320 (30% *v*/*v*). (**B**) Merger of red (actin) and green (spectrin) channels of the confocal microscope after examination of spectrin–actin double stained erythrocytes suspended in a mixture of plasma and Iopromide370 (30% *v*/*v*). (Primary magnification 1:63; zoom factor 3.5).

Supplementation of autologous plasma with RCM and suspension of erythrocytes in the RCM/plasma mixture induced—especially in the case of Iopromide—considerable alterations in the membrane cytoskeleton in comparison to erythrocytes suspended in an Iodixanol/plasma mixture [20]. There were practically no differences between erythrocytes suspended in autologous plasma (Figure 5 and Figure 6) or in a plasma/Iodixanol mixture (Figure 7A) [20] (except a slightly enhanced tendency for erythrocyte aggregation compared to erythrocytes suspended in autologous plasma).

Figure 7B demonstrates that on one hand there was a clear dissociation of red and green stained components in central cell parts and on the other hand there was a marked association of spectrin and actin components at the cell rim coinciding with a spectrin–actin co-localization revealed by the appearance of light yellow-green color bands.

Plasma supplemented with Iopromide370 (30% *v*/*v*) induced changes of the erythrocyte membrane which can be described as rounded bubble-like protrusions (see red protrusions in Figure 7B). Acuate spicules—as described in light-microscopic or REM images—could not be observed here. The actin–spectrin double staining revealed that the well rounded protrusions were (at least) coated with actin.

**Figure 8:**
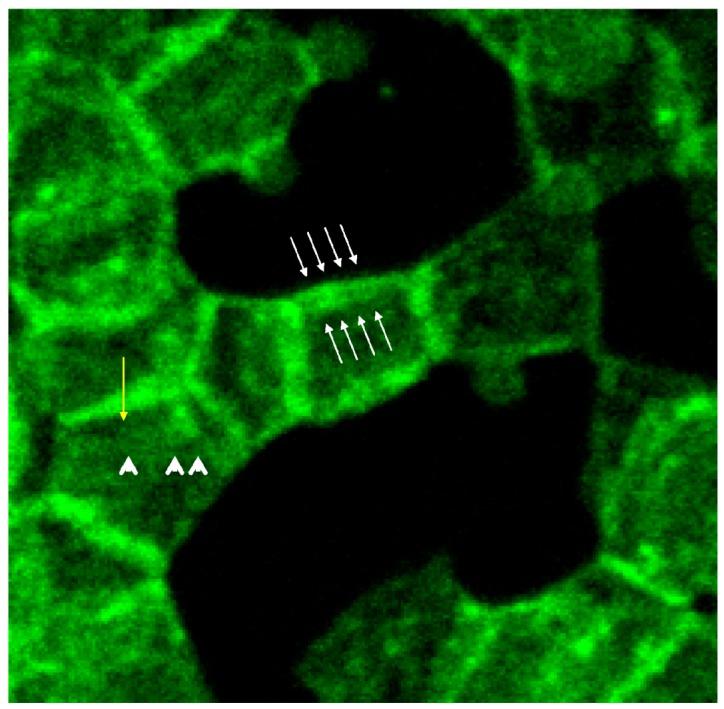
Spectrin stained erythrocytes suspended in a plasma/Iopromide 370 mixture (reprinted from reference [20], Copyright 2013, with permission from IOS Press; primary magnification 1:63; zoom factor 4.5).

White arrows (see Figure 8) mark neighbored double bands of spectrin at the cell rim also showing more intensely stained knob-like structures with spectrin bands [20]. In central parts of the erythrocyte also thin spectrin filaments (see arrow heads) are recognizable as well as singularized knobs of spectrin (see yellow arrow).

The box-like arrangement of the membrane cytoskeleton was often rectangularly deformed mainly showing spectrin and co-localized actin which can be deduced from the light yellow-green colored bands. Co-localized spectrin–actin bands were visible also at the membrane roots of the protrusions. Such membrane-anchored actin rings are assumed to support contractile forces in different somatic cell types to enact the exocytotic process [37].

**Figure 9:**
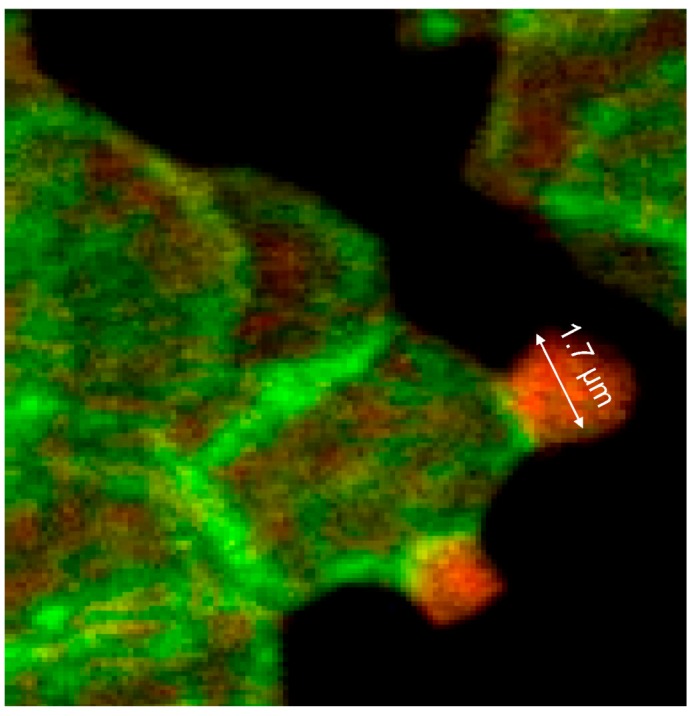
Merger of the red (actin) and green (spectrin) channels of the confocal microscope after examination of double-stained erythrocytes after incubation in a plasma/Iopromide370 mixture (reprinted from reference [20], Copyright 2013, with permission from IOS Press; primary magnification 1:63; zoom factor 5).

Thick bundles of long actin filaments at least coated the protrusions (see Figure 9; diameter: 1.7 µm) which initially were oriented perpendicular to the membrane. This could indicate that the schematic representation of the membrane cytoskeleton given by Drenckhahn [28] in which actin was arranged perpendicularly to the cell membrane seems to be closer to reality than schematic drawings where actin was arranged in parallel to the membrane.

At the membrane root of the protrusions circular spectrin–actin bands were found whose light yellow-green colors indicated co-localized spectrin and actin (see Figure 9).

**Figure 10:**
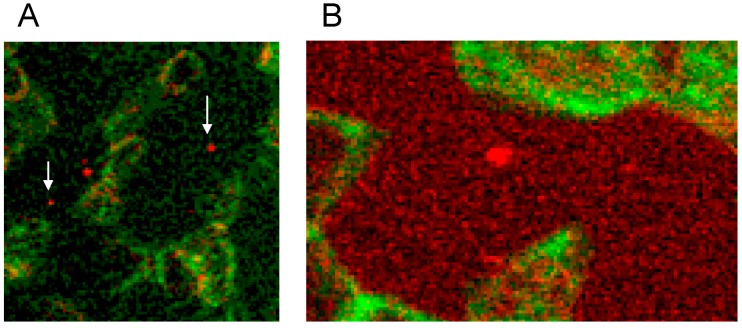
(**A**) Antibody stained actin particles outside erythrocytes marked by white arrows, Particles stained with antibodies against β-actin (reprinted from reference [22], Copyright 2014, with permission from IOS Press); (**B**) Particles stained with antibodies against band 3 (reprinted from reference [21]).

There was not only a different arrangement of actin in the membrane cytoskeleton of erythrocytes after incubation in different plasma/RCM mixtures. Actin oligomers could be found outside of erythrocytes mainly when cells were suspended in a plasma/Iopromide mixture (see Figure 10A, white arrows). Not only were actin oligomers found outside erythrocytes, but also band 3 oligomers, which had been described in a former study (see Figure 10B) [21].

This demonstrated that RCM can not only induce echinocyte formation but seemingly induce also an exocytosis-like process to export cell contents from the inside of erythrocytes to the outside.

**Figure 11:**
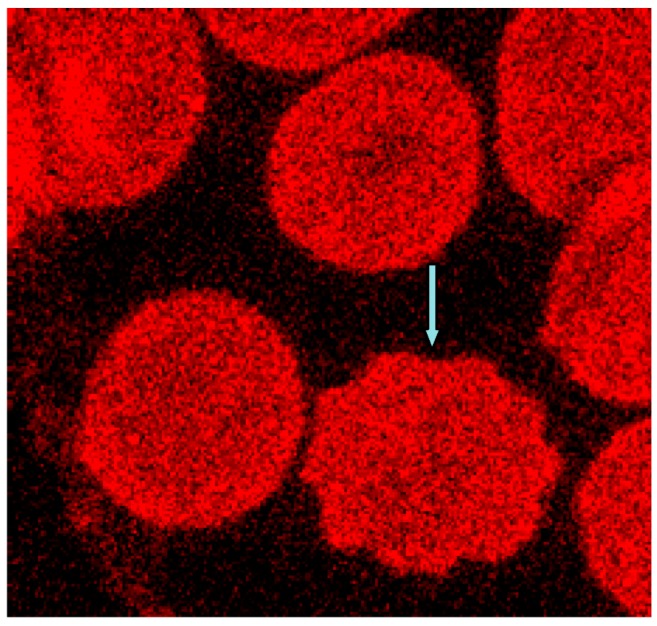
Phalloidin–Rhodamin stained erythrocytes, echinocytic shape change marked by the blue arrow (reprinted from reference [23], Copyright 2013, with permission from IOS Press; primary magnification 1:63, zoom factor: 5).

In the immunofluorescence, the membrane cytoskeleton of many cells was presented in a more or less well rounded conformation, although in the case of erythrocyte contact with Iopromide about 80% of erythrocytes were transformed to echinocytes [6]. Figure 11 shows (see blue arrow) the echinocytic transformation of the membrane cytoskeleton.

Shape changes of the erythrocyte cell membrane—visualized by light microscopy—are thought to coincide with the uncoupling of links between cell membrane and membrane cytoskeleton. There is a high number of these links in the well rounded erythrocytes suspended in autologous plasma, but there are considerably less numbers of these links in erythrocytes suspended in an Iopromide/plasma mixture where the actin/spectrin network is box-like arranged [20] while the cell membrane is rounded, somehow carrying also small and large more or less rounded protrusions. There are different types of links between cell membrane and the membrane skeleton. Looking at the results presented, there have to be elements in the membrane cytoskeleton, which can execute local shape changes *in vitro* and in the absence of external shear forces. These elements have to expend the energy on the local deformation work. One of these structures engaged in coupling of the membrane cytoskeleton to the cell membrane and through the positioning of its actin component—perpendicular to the cell membrane—suited to exert local shape changes, is the junctional complex. The membrane bilayer together with the membrane-associated proteins binding the cytoskeleton were described to regulate the characteristic shape, the membrane stability as well as the elastic properties of erythrocytes [19]. The core of the cytoskeleton consists of, e.g., spectrin, actin, adducin, band 4.1 and band 4.9 (also known as dematin [38]). Dematin—located at the vertices of the mostly hexagonal spectrin filament network [38]—is a component of the junctional complex recently shown to anchor the tail-end of spectrin to the glucose transporter 1 (GLUT1) in the erythrocyte membrane [17,18,39]. In addition, dematin is a potent actin bundling protein *in vitro* and probably also *in vivo* [40] where actin-binding sites were assumed to be contained in the headpiece domain and in the intrinsically disordered core domain [34,40].

**Figure 12:**
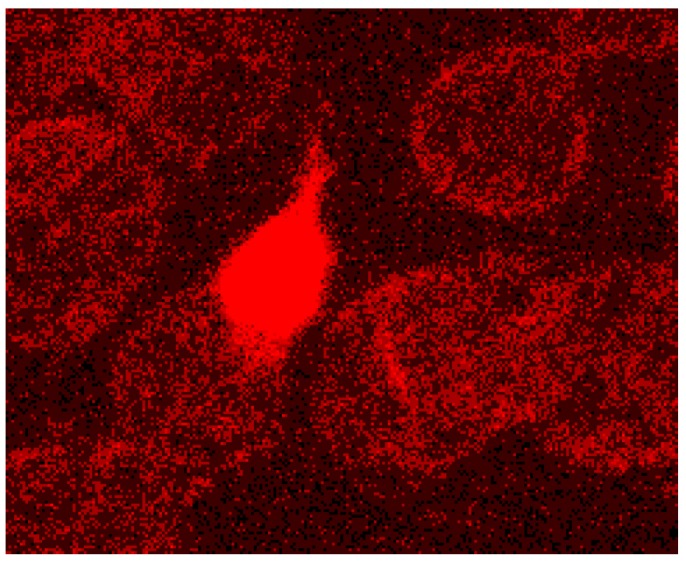
Protrusion, at least coated by bundled actin filaments, with a protrusion length of nearly the complete cell diameter (reprinted from reference [23], Copyright 2013, with permission from IOS Press).

In addition the polymerization of actin is thought to be necessary leading to a considerable lengthening of the actin oligomers. In discocytes long actin filaments were shown recently whose length had increased and exceeded 1/3 of the cell diameter [20] (see Figure 9 and Figure 12).

**Figure 13:**
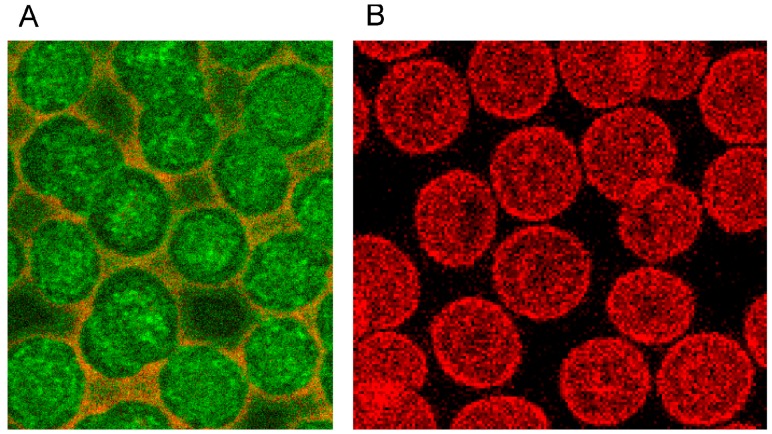
(**A**) Band 4.9—stained erythrocytes after suspension in autologous plasma (**B**) Phalloidin–Rhodamin stained actin in erythrocytes after suspension in autologous plasma ((**A**) reprinted from reference [22], Copyright 2014, with permission from IOS Press; (**A**) reprinted from reference [23], Copyright 2013, with permission from IOS Press; primary magnification 1:63; zoom factor: 5).

The distribution of band 4.9 in the cytoskeleton of erythrocytes is shown in Figure 13. A knob-like structure and a less homogenous distribution of band 4.9 in erythrocytes suspended in autologous plasma were found. In most cells an almost unstained, circular area appeared in the rim of the cells surrounded by a peripheral narrow green-stained band (see Figure 13A). While actin usually prevailed in the strongly curved cell regions (see Figure 13B), band 4.9 appeared also in the central regions of erythrocytes. This gave for the first time evidence that band 4.9 is not only associated with actin but possibly also with other elements in the cell center which might serve as a band 4.9 depot. It might be hypothesized that such a depot enables exocytotic processes or fast shape changes of erythrocytes.

**Figure 14:**
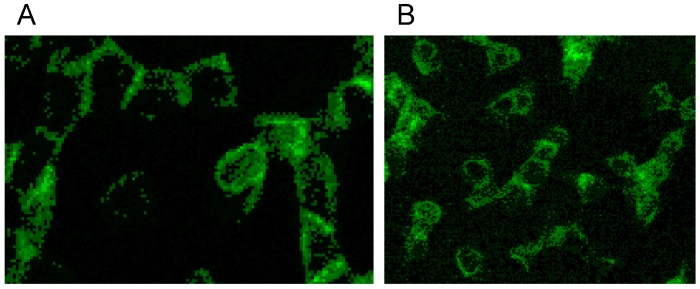
Band 4.9 stained erythrocytes after suspension of erythrocytes in (**A**) an Iodixanol/plasma mixture or (**B**) in an Iopromide/plasma mixture (RCM 30% *v*/*v*) (reprinted from reference [23], Copyright 2013, with permission from IOS Press).

Both RCM translocated band 4.9 out of central cell regions and into the rim of erythrocytes (see Figure 14A,B). While incubation of erythrocytes in an Iodixanol/plasma mixture induced relatively thick bands with great numbers of knob-like structures at the cell rim (see Figure 14A), the incubation in an Iopromide/plasma mixture induced a formation of thin band 4.9 bands with only few knob-like structures at the cell rim and with cell bodies almost completely devoid of band 4.9 (see Figure 14B).

**Figure 15:**
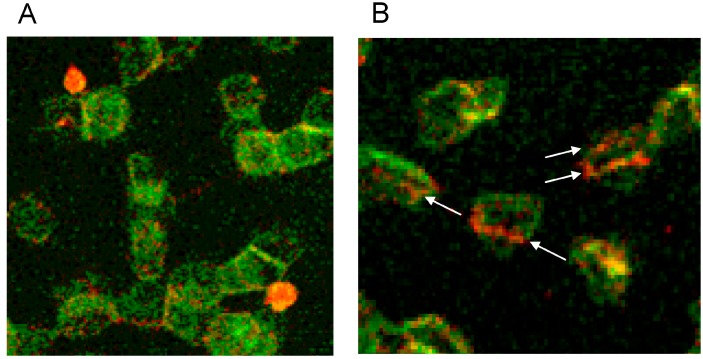
(**A**) Antibody stained Actin protrusions in erythrocytes suspended in a plasma/Iodixanol mixture (30% *v*/*v*) or (**B**) Band 4.9 bands in erythrocytes in a plasma/Iopromide mixture (30% *v*/*v*) (reprinted from reference [23], Copyright 2013, with permission from IOS Press).

Double staining of actin and band 4.9 revealed that only in erythrocytes suspended in an Iopromide/plasma mixture bands of actin appeared—probably through actin polymerization and bundling—passing through the whole cells (see white arrows in Figure 15B).

**Figure 16:**
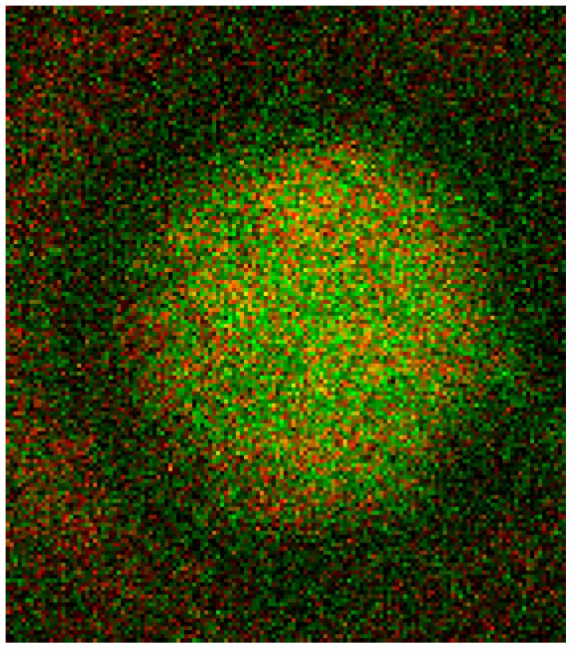
Merger of red (band 3) and green (spectrin) channels of the confocal microscope after examination of double stained erythrocytes suspended in autologous plasma (reprinted from reference [21]; Primary magnification: 1:63; zoom factor 5).

The junctional complex is coupled to the erythrocyte membrane via band 3 [27]. Band 3 is found in three distinct protein complexes in erythrocyte membrane: an ankyrin-dependent tetrameric band 3 complex, a dimeric band 3 complex bound to the protein 4.1-glycophorin C junctional complex and a freely diffusing dimeric band 3 complex. The distribution of band 3 in erythrocytes suspended in autologous plasma is shown in Figure 16 after double staining with antibodies against spectrin and band 3. Spectrin and band 3 were practically distributed homogenously. Co-localizations of spectrin and band 3 were not observed.

**Figure 17:**
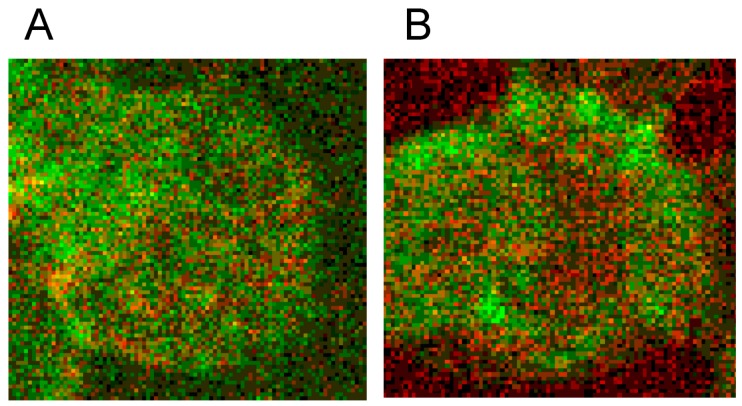
Merger of red (band 3) and green (spectrin) channels of the confocal microscope after examination of double stained erythrocytes suspended in RCM/plasma mixtures (reprinted from reference [21]). (**A**) Erythrocyte suspended in Iodixanol/plasma mixture (30% *v*/*v*); (**B**) Erythrocyte suspended in Iopromide/plasma mixture (30% *v*/*v*). (Primary magnification: 1:63; zoom factor 5).

Different from strong changes in the distribution of band 4.9, there were only weak changes in the distribution of band 3 after erythrocyte suspension in an Iodixanol/plasma mixture (see Figure 17A). Changes in band 3 localization were remarkable after erythrocyte contact with Iopromide. A condensation of knoblike structures at the cell rim was observed coinciding with a strong decrease in stain intensity in the cell center and a slight increase of band 3 and spectrin co-localization (yellow shift) in the knob-like structures (see Figure 17B).

Well rounded and larger invaginations appeared in the cell membrane resembling the formation of caveolae in some cells as well as slender, narrow and very pointed protrusions of the cell membrane to the outside [26]. These big differences in the shape change of erythrocytes are possibly due to different effective mechanisms.

**Figure 18:**
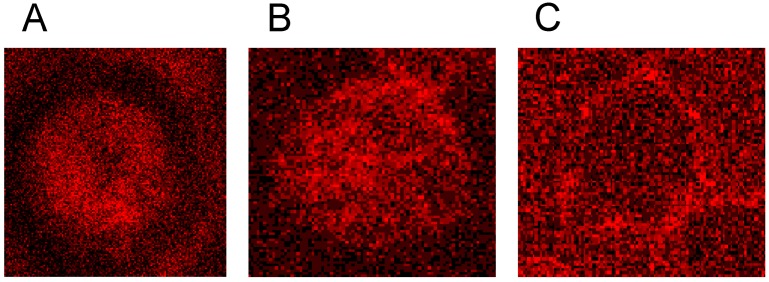
Representative erythrocytes stained with antibodies against band 3 (reprinted from reference [21]). (**A**) erythrocyte suspended in autologous plasma; (**B**) erythrocyte suspended in Iodixanol/plasma mixture (30% *v*/*v*); (**C**) erythrocyte suspended in Iopromide/Plasma mixture (30% *v*/*v*) (Primary magnification: 1:63; zoom factor 5).

Band 3 is described to constitute a center organized into complexes for gas transport [41,42,43,44], anion exchanger, for glycolysis [43,45,46], for the control of cell volume [47] and of erythrocyte life span (senescence) [48], where most probably further complexes exist to cope with mechanical deformation [49], and haemostatic stimuli [50,51]. The drastic reorganization of the band 3—spectrin network affected by suspension of erythrocytes in an Iopromide/plasma mixture coincided with a strong alteration of the spectrin network and a clustering of band 3 in very few highly condensed centers at the rim of the cells (see Figure 18). This is most probably accompanied by a strong increase in membrane stiffness (contributing to microcirculatory disorders shown in patients with coronary artery disease after Iopromide application during coronary angiography [10]). Together with translocation of band 3 particles out of erythrocytes, a strong decrease of centers for gas transport can be expected, contributing to hypo-oxygenation of the tissue, as was shown after Iopromide application in the myocardium of pigs [12] but not after Iodixanol application [52]. The clustering of band 3 is assumed to lead to a shortening of the lifespan and removal of erythrocytes by offering senescence signals. The remarkable loss of band 3, revealed by a sequestration of band 3 out of the tips of the spicules, is thought to give clear evidence that the echinocyte formation provoked by RCM is associated with an exocytotic process—a mechanism which was not described up to now.

## 7. Conclusions

RCM induced—beside the well-known and often demonstrated echinocyte formation—a clear alteration of the membrane cytoskeleton. In four co-localization studies we could demonstrate for the first time that Iopromide induced a rectangularly arranged spectrin configuration completely different from the spectrin network in erythrocytes in autologous plasma or in an Iodixanol/plasma mixture. The Iopromide-induced box-like rectangular arrangement of the spectrin configuration is composed of thick spectrin bands which evidently afford loosening of many linkages between the cytoskeleton and the membrane and which is coincident with a completely different distribution of band 3, now mostly found in few knob-like structures. The bundling of band 3 alone could lead to a loss of function of the band 3 complex and therefore of the whole cell because it might restrict the exchange of breathing gas and anions. Iopromide also altered the distribution of band 4.9 drastically (from a more or less homogeneous distribution with knobs to a banded distribution concentrated at the cell rim), which seemed to enable or even foster the box-like arrangement of the membrane cytoskeleton. Since band 4.9 is an element of the junctional complex linking the membrane cytoskeleton to the membrane this is a clear indicator that a considerable number of links is lost allowing for a transition of the spheroid into the rectangular configuration of the membrane cytoskeleton.

In addition, Iopromide influenced the actin polymerization seriously: After contact of erythrocytes with Iopromide extremely long actin filaments appeared (up to the complete cell diameter) thus crossing the whole cell, which never was observed in autologous plasma or after contact with Iodixanol. This could possibly be caused by the altered distribution of band 4.9 which is a prominent actin capping and bundling protein. The band 4.9, now not longer ubiquitously available and concentrated at the cell rim, could cause the strong tendency to generate long-chain actin filaments especially in central parts of the erythrocytes.

After suspension of erythrocytes in a plasma/Iodixanol mixture an increased number of membrane protrusions oriented perpendicular to the cell membrane appeared, a process which also appeared in cells suspended in autologous plasma, however, in fewer numbers. Suspension in Iopromide, in contrast, induced a complete reorganization of the cytoskeletal actin: the fine grained globular actin distribution disappeared and only few, long and thick actin filaments bundled and possibly polymerized appeared, instead, shown here for the first time. It was shown that the particles—evidently exported from erythrocytes—were at least coated with actin and/or band 3.

Iopromide, evidently, induced markedly more severe alterations of the membrane skeleton compared to Iodixanol whose effects were similar to erythrocytes suspended in autologous plasma.
